# *Trichuris muris* whey acidic protein induces type 2 protective immunity against whipworm

**DOI:** 10.1371/journal.ppat.1007273

**Published:** 2018-08-28

**Authors:** Neima Briggs, Junfei Wei, Leroy Versteeg, Bin Zhan, Brian Keegan, Ashish Damania, Jeroen Pollet, Kelly S. Hayes, Coreen Beaumier, Christopher A. Seid, Jamie Leong, Richard K. Grencis, Maria Elena Bottazzi, K. Jagannadha Sastry, Peter J. Hotez

**Affiliations:** 1 Texas Children’s Hospital Center for Vaccine Development, Department of Pediatric Tropical Medicine, National School of Tropical Medicine, Baylor College of Medicine, Houston, TX, United States of America; 2 MD Anderson Cancer Center UTHealth Graduate School of Biomedical Sciences, Houston, Texas, United States of America; 3 Department of Immunology, The University of Texas M.D. Anderson Cancer Center, Houston, Texas, United States of America; 4 School of Biological Sciences, FBMH, MAHSC, University of Manchester, Manchester, United Kingdom; 5 Wellcome Trust Centre for Cell Matrix Research, University of Manchester, Manchester, United Kingdom; 6 The Lydia Becker Institute for Immunology and Inflammation, University of Manchester, Manchester, United Kingdom; 7 Department of Biology, Baylor University, Waco, Texas, United States of America; Uniformed Services University of the Health Sciences, UNITED STATES

## Abstract

Human whipworm (*Trichuris trichiura*) infects approximately 1 in 15 people worldwide, representing the leading infectious cause of colitis and subsequent, inflammatory bowel disease (IBD). Current control measures focused on mass deworming have had limited success due to low drug efficacies. Vaccination would be an ideal, cost-effective strategy to induce protective immunity, leading to control of infection and transmission. Here we report the identification of whey acidic protein, a whipworm secretory protein, as a strong immunogen for inducing protective efficacy in a surrogate mouse *T*. *muris* infection model. The recombinant WAP protein (r*Tm*-WAP49), as well as a single, highly conserved repeat within WAP (fragment 8) expressed as an *Na-*GST-1 fusion protein (r*Tm*-WAP-F8+*Na*-GST-1), generate a strong T helper type 2 (Th2) immune response when delivered as subcutaneous vaccines formulated with Montanide ISA 720. Oral challenge with *T*. *muris* infective eggs following vaccination led to a significant reduction in worm burden of 48% by r*Tm*-WAP49 and 33% by r*Tm*-WAP-F8+*Na*-GST-1. The cellular immune correlates of protection included significant antigen-specific production of Th2 cytokines IL-4, IL-9, and IL-13 by cells isolated from the vaccine-draining inguinal lymph nodes, parasite-draining mesenteric lymph nodes, and spleen in mice vaccinated with either r*Tm*-WAP49 or r*Tm*-WAP-F8+*Na*-GST-1. The humoral immune correlates included a high antigen-specific ratio of IgG1 to IgG2a, without eliciting an IgE-mediated allergic response. Immunofluorescent staining of adult *T*. *muris* with WAP antisera identified the worm’s pathogenic stichosome organ as the site of secretion of native *Tm*-WAP protein into the colonic mucosa. Given the high sequence conservation for the WAP proteins from *T*. *muris* and *T*. *trichiura*, the results presented here support the WAP protein to be further evaluated as a potential human whipworm vaccine candidate.

## Author summary

Human whipworm (*Trichuris trichiura*) is pervasive intestinal parasite that causes malnutrition and inflammatory bowel disease (IBD), predominately among the world’s most impoverished billion people. Available treatment strategies have had little impact on the disease prevalence or morbidity. A vaccine against human whipworm would provide a low-cost and long-term solution, yet the identification of candidates is lacking. Here we characterize a stichosome-derived whey acidic protein (WAP), dominant among the worm’s excretory and secretory (ES) products, as a potential vaccine candidate. When delivered as a subcutaneous recombinant protein formulated with the clinically relevant adjuvant, Montanide ISA 720, mice were significantly protected against challenge infection. We describe the induced protection as a combined WAP-specific type 2 cellular and humoral immune response. In summary, we have identified an immunogenic whipworm protein that is effective in inducing protective efficacy in an animal model and is a promising human vaccine candidate.

## Introduction

It is estimated that 465 million people are living with whipworms (*Trichuris trichiura*) in their colon, predominately in the developing nations of the Americas, Asia, and Africa. In children, *T*. *trichiura* infections cause chronic colitis, resulting in nutritional deficiencies, growth stunting, and cognitive impairment [1]. In adults, *Trichuris*-induced colitis is being studied as a possible major environmental driver of inflammatory bowel disease [2]. The current approach to human trichuriasis control relies on mass deworming campaigns with yearly, single dose albendazole or mebendazole [3]. However, this approach has shown limited benefit against trichuriasis due to low efficacy, high rates of post-treatment reinfection, and failure to co-implement adequate sanitation and hygiene practices [4,5]. It is therefore important to design better or alternative strategies to helminth prevention, including the development of a trichuriasis vaccine [6]. While there is no direct evidence that humans develop protective immunity to trichuriasis, epidemiological studies suggest that there is an age-related reduction in worm burden and adult worm longevity in humans beginning in adolescence that may be due to acquired immunity [7,8].

The closely related *Trichuris muris* parasite, specific to mice, is a well-established model for disease pathophysiology and subsequent host immunity, and is widely used for evaluating the immunogenicity and efficacy of vaccine candidates [9,10]. In both mice and humans, ingested whipworm eggs hatch into larvae in the terminal ileum and caecum due to interactions with the host microbiome [11]. There, the helminths’ anterior-end organ, known as a stichosome, excretes products implicated in facilitating the invasion and maintenance of the parasite into the host’s gut epithelia [9,12]. Once embedded, the *Trichuris* worms become fixed in location, causing inflammation at the site of intracellular attachment and induce colitis-like pathology [9]. The *T*. *muris* excretions and secretions (ES), thought to predominately originate from the stichosome, can be collected from the media of *in-vitro* cultured helminths. We and others have reported that vaccination with *T*. *muris* ES product elicits protective immunity in murine models [13–15]. Protective immunity in preclinical models correlates with T helper type 2 (Th2) responses, characterized by the production of cytokines IL-4, IL-9, and IL-13 along with IgG1 antibodies and high IgG1 to IgG2a ratios [9,13,15–18]. Conversely, susceptibility is characterized by a predominate TH1 response (IL-12 and Interferon (IFN)-γ) and induction of IgG2a antibodies [9,13,16].

Here we report the identification and analyses of immunogenicity as well as efficacy of two highly abundant secretory proteins, whey acidic protein (WAP) and cysteine-rich secretory proteins (CRISPS), Antigen 5, and Pathogenesis-related 1 (CAP-1), from among the *T*. *muris* ES antigens. Recombinant *Tm*-WAP (r*Tm*-WAP49) induced significant protection against *T*. *muris* challenge, while recombinant *Tm*-CAP-1 was ineffective. Protection against *T*. *muris* infection by r*Tm*-WAP49 was associated with strong Th2 cellular and humoral immune responses.

## Results

### Immunoscreening for the identification of highly abundant *T*. *muris* excreted-secreted (ES) antigens

**Fig 1:**
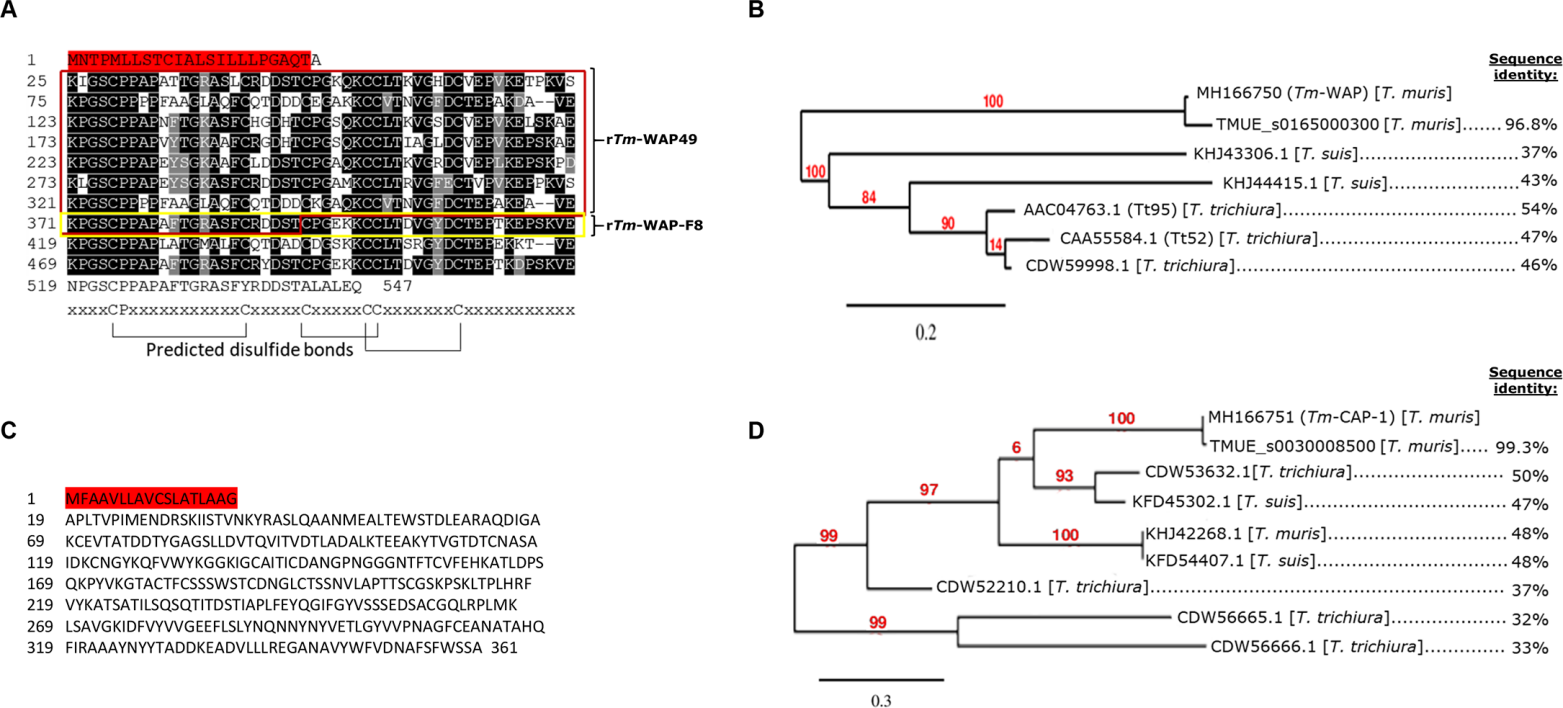
Amino acid sequences and phylogenetic analyses of the WAP and CAP-1 proteins from *T*. *muris*. The predicted amino acid sequences of (A) *Tm*-WAP and (C) *Tm*-CAP-1. The predicted signal peptide of each protein is highlighted in red (SignalP 4.1). The *Tm*-WAP protein contains ten repeats of a whey acidic protein (WAP)-type four-disulfide core domain that were aligned using CLUSTAL W and prepared for display using BOXSHADE. Identical amino acids are shaded in black and similar amino acids in gray. This 50 amino acid domain contains 6 conserved cysteine residues. The 49 kDa N-terminus sequence was expressed in yeast as r*Tm*-WAP49 (in red box). The repeat fragment #8 was expressed in *E*. *coli* as *Tm*-WAP-F8 (in yellow box) using an *Na*-GST-1 expression tag. Phylogenetic tree depicting the sequence identify of (B) *Tm*-WAP and corresponding gene TMUE_s0165000300 and (D) r*Tm*-CAP-1 and corresponding gene TMUE (s0030008500), to their respective homologues in different *Trichuris* species.

Serum from mice immunized with *T*. *muris* ES product was used to screen the *T*. *muris* adult whole worm expressional cDNA library and identified a total of 102 positive clones. DNA sequencing revealed a whey acidic protein (*Tm*-WAP) as the most abundant of the candidates identified (63 clones). BLAST searching revealed that *Tm*-WAP shares 96.8% amino acid sequence identity with a gene product (TMUE_s0165000300) in the established *T*. *muris* genome on WormBase [19,20]. The *Tm*-WAP protein consists of several repeats (3–10) of a 50 amino acid fragment that share 69–96% sequence identity and possess six conserved cysteines predicted to form multiple disulfide bonds (**Fig 1A**) (DiANNA 1.1) [21–23]. The *Tm*-WAP gene shares high sequence alignment with the *T*. *trichiura* putative porin proteins, TT95 [24] and TT52 [25], at 54% and 47% amino acid sequence identity, respectively (**Fig 1B**).

The second most abundant clone identified from the cDNA library screening was *T*. *muris* cysteine-rich secretory proteins (CRISPS), Antigen 5, and Pathogenesis-related 1 (CAP-1) protein (35 clones) (**Fig 1C**). The CAP-domain family (also known as SCP/TAPS) was previously identified based on its abundance in the secretome of whipworm and other soil-transmitted helminths [26,27]. BLAST searching revealed that *Tm*-CAP-1 shares 99.3% amino acid sequence identity with a gene product (TMUE_s0030008500) in the established *T*. *muris* genome on WormBase [19,20]. *Tm*-CAP-1 shares the highest sequence alignment with the *T*. *suis* SCP-like protein (accession number KHJ42268.1) and *T*. *trichiura* CAP-domain containing protein (accession number CDW52210.1), at 48% and 38% amino acid sequence identity, respectively (**Fig 1D**).

High sequence alignment (defined as >40%) is strongly predictive of protein homology with shared structure [28]. This suggests that both WAP and CAP-1 would be translatable targets of a future *T*. *trichiura* vaccine.

The *Tm*-WAP protein was expressed with a 6His-tag at C-terminus in *Pichia pastoris* X33 as a soluble protein and purified by IMAC. After multiple unsuccessful attempts at expressing the entire protein in *E*. *coli*, ultimately yeast were found to be an efficient expression platform. The most conserved repeat of *Tm*-WAP, fragment 8, was expressed with a *Necator americanus* glutathione s-transferase-1 (*Na*-GST-1)*-*tag at the N-terminus as a soluble recombinant fusion protein in *E*. *coli* BL21 under induction of 1 mM IPTG and purified with immobilized IMAC. In addition to improving the expression and solubility of the fusion protein, *Na*-GST-1 is a hookworm vaccine candidate [29], and selected for the potential as a pan-anthelminthic vaccine candidate. The recombinant protein migrated at 49 kDa, therefore named r*Tm*-WAP49. The full-length recombinant *Tm*-CAP-1 protein was expressed in *Escherichia coli (E*. *coli)* BL21 as an insoluble inclusion body and solubilized in 8M urea. The r*Tm*-CAP-1 with an 8 his-tag at C-terminus was purified with immobilized metal affinity chromatography (IMAC), as previously described [30].

### Analyses of immunogenicity and protective efficacy of the *T*. *muris* proteins selected from immunoscreening

While the expression and purification of r*Tm*-WAP49 was in progress, we evaluated the immunogenicity of r*Tm*-WAP-F8+*Na-*GST-1 and r*Tm*-CAP-1 in the AKR mouse model. We selected the AKR mouse model because these mice develop a type 1 immune response to *T*. *muris* challenge alone, leading to a high-burden of disease, yet retain the ability to develop a protective type 2 immune response if vaccinated with *Tm*-ES prior to challenge [13].

**Fig 2:**
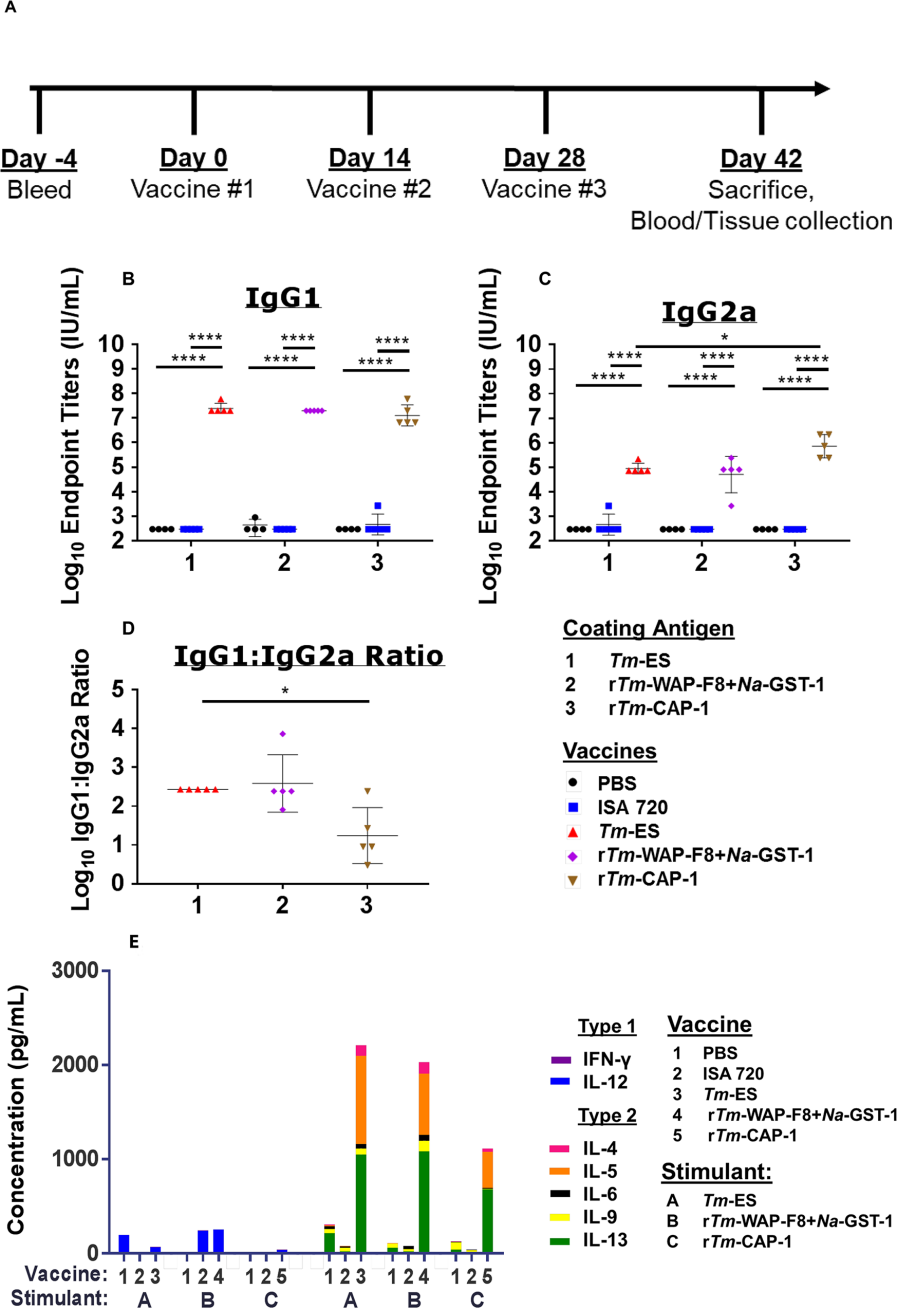
Immunogenicity of r*Tm*-WAP-F8+*Na*-GST-1 and r*Tm*-CAP-1 proteins in a murine model. (A) Vaccination schedule to evaluate immunogenicity in male AKR/J mice. Humoral immune response was measured by end-point serum titer for antigen-specific (B) IgG1 and (C) IgG2a by ELISA to calculate the (D) IgG1 to IgG2a ratio for the mice in each group. Mice in groups injected with PBS or Montanide ISA 720 adjuvant were used as negative controls for each coating antigen. Statistical significance: *p<0.05, **p<0.01 ***p<0.001, ****p<0.0001. (E) Cellular immune responses in terms of cytokines in the supernatants of splenocytes stimulated with media or cognate antigens, r*Tm*-WAP-F8+*Na*-GST-1 (10 μg/mL), r*Tm*-CAP-1 (10 μg/mL), or *Tm*-ES (50 μg/mL), for 72 hours. Values for cells cultured in media only were subtracted as background from those of cells stimulated with cognate antigens in the corresponding groups of mice. Values from duplicate wells for each treatment/stimulation were averaged for individual cytokines for the 5 mice in each group to calculate statistical significance. Individual cytokines and statistics are shown in S1 Fig.

Separate groups of mice were immunized three times at two-week intervals with *Tm*-ES, r*Tm*-WAP-F8+*Na-*GST-1 or r*Tm*-CAP-1 using the Montanide ISA 720 adjuvant (**Fig 2A**). Montanide ISA 720 is a squalene-based water-in-oil emulsion used in multiple efficacious helminth vaccine animal studies, including the closely related *Trichinella spiralis*, and has demonstrated safety in humans [31–33]. Control groups included mice injected with PBS or adjuvant alone. Two weeks after the final immunization, serum levels of antigen-specific IgG1 and IgG2a were determined by ELISA to calculate end-point titers. Mice immunized with *Tm*-ES, r*Tm*-WAP-F8+*Na-*GST-1, or r*Tm*-CAP-1 exhibited significant levels of respective antigen-specific IgG1 as well as IgG2a titers relative to those in control groups of mice immunized with PBS or adjuvant ISA-720 (**Fig 2A and 2B**). However, antigen-specific IgG2a levels were significantly higher in mice immunized with r*Tm*-CAP-1 relative to those in mice immunized with *Tm*-ES (**Fig 2C**). Consequently, mice immunized with r*Tm*-CAP-1 had a significantly lower IgG1:IgG2a ratio relative to those immunized with either *Tm*-ES or r*Tm*-WAP-F8+*Na-*GST-1 (**Fig 2D**). Next, an analysis of antigen-specific T cell response of mice immunized with the different antigens was conducted. Spleen cells were isolated and restimulated with the cognate proteins for 72 hours *in vitro*. In comparison to the mice in the two negative control groups (PBS or adjuvant control) cognate antigen-specific T cell responses, in terms of type 2 cytokine production, were significantly higher in mice immunized with *Tm*-ES, r*Tm*-WAP-F8+*Na-*GST-1, and r*Tm*-CAP-1 (**Fig 2E**). However, the production of type 1 cytokines IFN-γ and IL-12 in splenocytes was low and comparable between mice in the different vaccine groups, as well as the adjuvant control group. Importantly, production of type 2 cytokines IL-4 and IL-13 was significantly higher in mice immunized with either r*Tm*-WAP-F8+*Na-*GST-1 or *Tm*-ES compared to that in mice immunized with r*Tm*-CAP-1 (**S1 Fig**).

**Fig 3:**
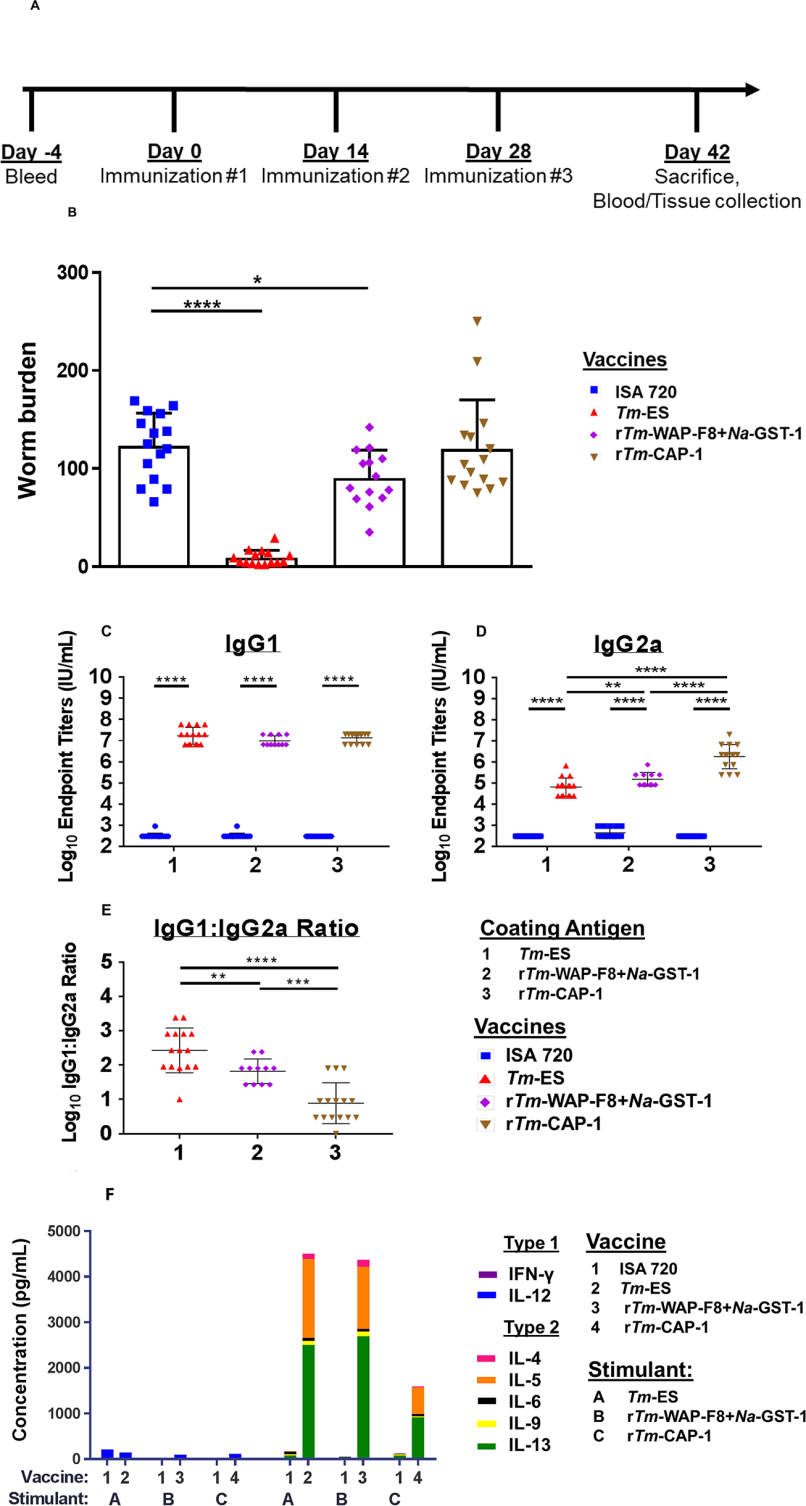
Efficacy and anamnestic responses in mice immunized with r*Tm*-WAP-F8+*Na*-GST-1 and r*Tm*-CAP-1 proteins. (A) Vaccine schedule to measure efficacy and post-challenge anamnestic response in male AKR/J mice. (B) Vaccine efficacy was evaluated on day 15 post-infection by counting the numbers of worms in the intestines by microscopy. At time of sacrifice, anamnestic humoral response was measured by end-point serum titer for antigen-specific (C) IgG1 and (D) IgG2a by ELISA to calculate the (E) IgG1 to IgG2a ratio for the mice in each group. Mice in groups injected with Montanide ISA 720 were used as negative controls for each coating antigen. Statistical significance: *p<0.05, **p<0.01 ***p<0.001, ****p<0.0001. (F) Cellular immune responses in terms of cytokines in the supernatants of splenocytes stimulated with media or cognate antigen, r*Tm*-WAP-F8+*Na*-GST-1 (10 μg/mL), r*Tm*-CAP-1 (10 μg/mL), or *Tm*-ES (50 μg/mL), for 72 hours. Values from duplicate wells for each treatment/stimulation were averaged for individual cytokines for the 13–15 mice in each group to calculate statistical significance. Individual cytokines and statistics are shown in S2 Fig.

Mice vaccinated with r*Tm*-WAP-F8+*Na-*GST-1, r*Tm*-CAP-1, *Tm*-ES, or Montanide ISA 720 adjuvant alone were challenged orally with 300 embryonated *T*. *muris* eggs and evaluated 15 days post-infection for intestinal worm burden by direct microscopy (**Fig 3A**). In agreement with data from our earlier reported studies and those of others [13,34], mice vaccinated with *Tm*-ES exhibited a significant reduction (93%, p<0.0001) in mean worm burden compared to mice immunized with Montanide ISA 720 adjuvant alone (**Fig 3B**). Furthermore, vaccination with r*Tm*-WAP-F8+*Na*-GST-1 also induced significant protection (27% reduction in worm burden, p = 0.0124), while mice vaccinated with r*Tm*-CAP-1 showed only 3% reduction in worm burden (p = 0.845).

In addition to a strong humoral and cellular type 2 response generated by vaccination, a continued type 2 immune response is necessary during the “critical period” of worm expulsion (day 0–21 p.i.) [13]. We evaluated humoral and cellular immune responses post-challenge (anamnestic responses) by measuring the antigen-specific IgG1 and IgG2a levels in the serum along with T cell responses in splenocytes after restimulation *in vitro* with the cognate antigens. In mice vaccinated with *Tm*-ES and r*Tm*-WAP-F8+*Na*-GST-1, relative to adjuvant control group, we observed a significantly higher type 2 immune response, characterized by high IgG1:IgG2a ratios and T cells producing significantly higher levels of IL-4 and IL-13 in the spleen (**Fig 3C–3F**). These antibody and T cell responses were significantly lower in mice vaccinated with r*Tm*-CAP-1 when compared to those in mice vaccinated with *Tm*-ES or r*Tm*-WAP-F8+*Na*-GST-1. Furthermore, post-challenge, we observed T cells producing significant levels of IFN-γ in the spleens from mice immunized with r*Tm*-CAP-1 (**S2 Fig**).

### Recombinant WAP protein(r*Tm*-WAP49) induces strong type 2 protective immunity

**Fig 4:**
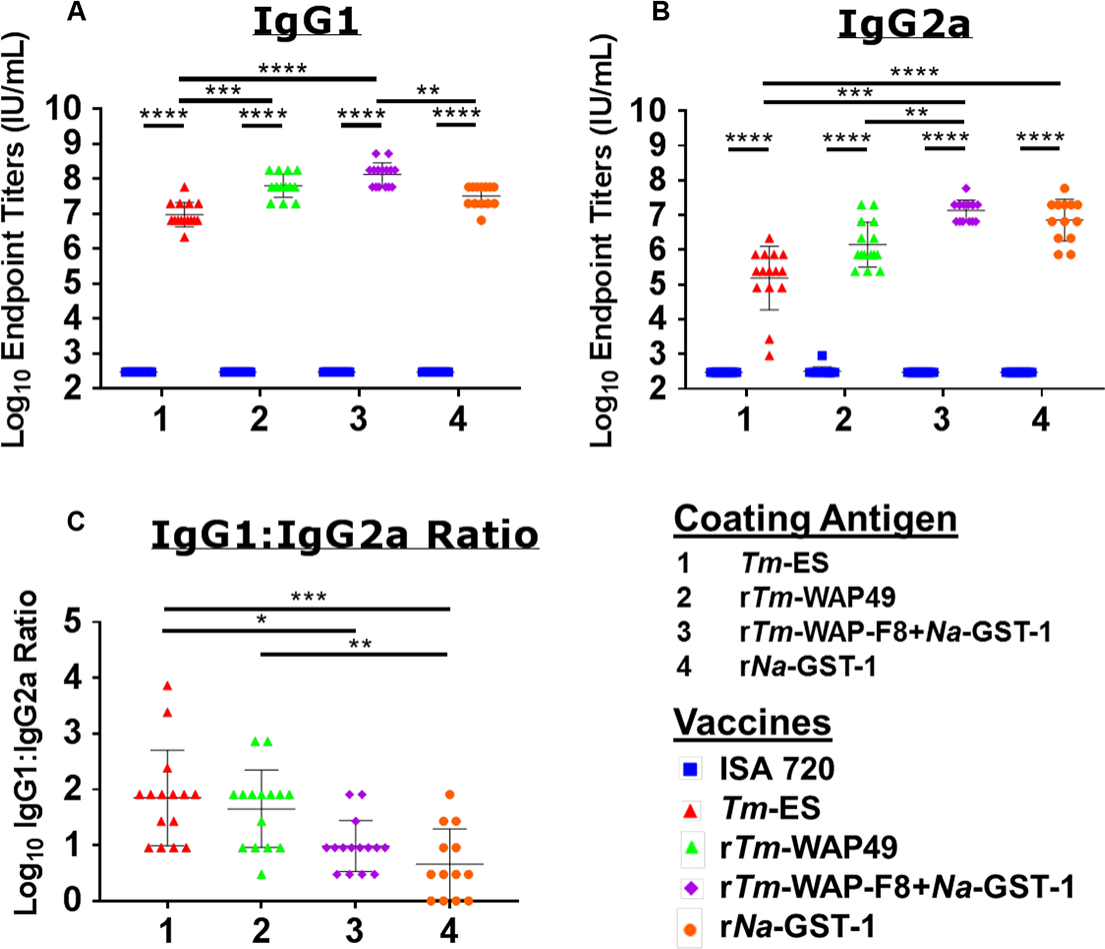
Immunogenicity of r*Tm*-WAP-F8+*Na*-GST-1 and r*Tm*-WAP49 proteins in a murine model. Humoral immune response was measured by end-point serum titer for antigen-specific (A) IgG1and (B) IgG2a by ELISA to calculate the (C) IgG1 to IgG2a ratio for the mice in each group. Mice in groups injected Montanide ISA 720 adjuvant were used as negative controls for each coating antigen. Values from duplicate wells for each treatment/stimulation were averaged for individual cytokines for the 15 mice in each group to calculate statistical significance. Statistical significance: *p<0.05, **p<0.01 ***p<0.001, ****p<0.0001.

The *Tm*-WAP protein, named r*Tm*-WAP49 expressed in *Pichia pastoris* X33 was used to evaluate immunogenicity and protective efficacy in comparison to that by the WAP fragment (r*Tm*-WAP-F8+*Na*-GST-1) following the immunization and challenge schedule shown in Fig 2A. To determine the contribution of the *Na*-GST-1 tag within the r*Tm*-WAP-F8+*Na*-GST-1, the recombinant *Na*-GST-1 protein was included as an additional control immunogen. Montanide ISA 720 adjuvant alone and *Tm*-ES were used as negative and positive control reagents, respectively. Endpoint titers of antigen-specific IgG1 (**Fig 4A**) and IgG2a **(Fig 4B**) in the serum samples rose significantly for all vaccine groups compared to adjuvant alone. While the cognate antigen-specific IgG1:IgG2a ratios in mice vaccinated with r*Tm*-WAP-F8+*Na*-GST-1 or r*Tm*-WAP49 were significantly higher compared to the adjuvant alone, these levels were lower relative to those in mice vaccinated with *Tm*-ES (**Fig 4C**).

**Fig 5:**
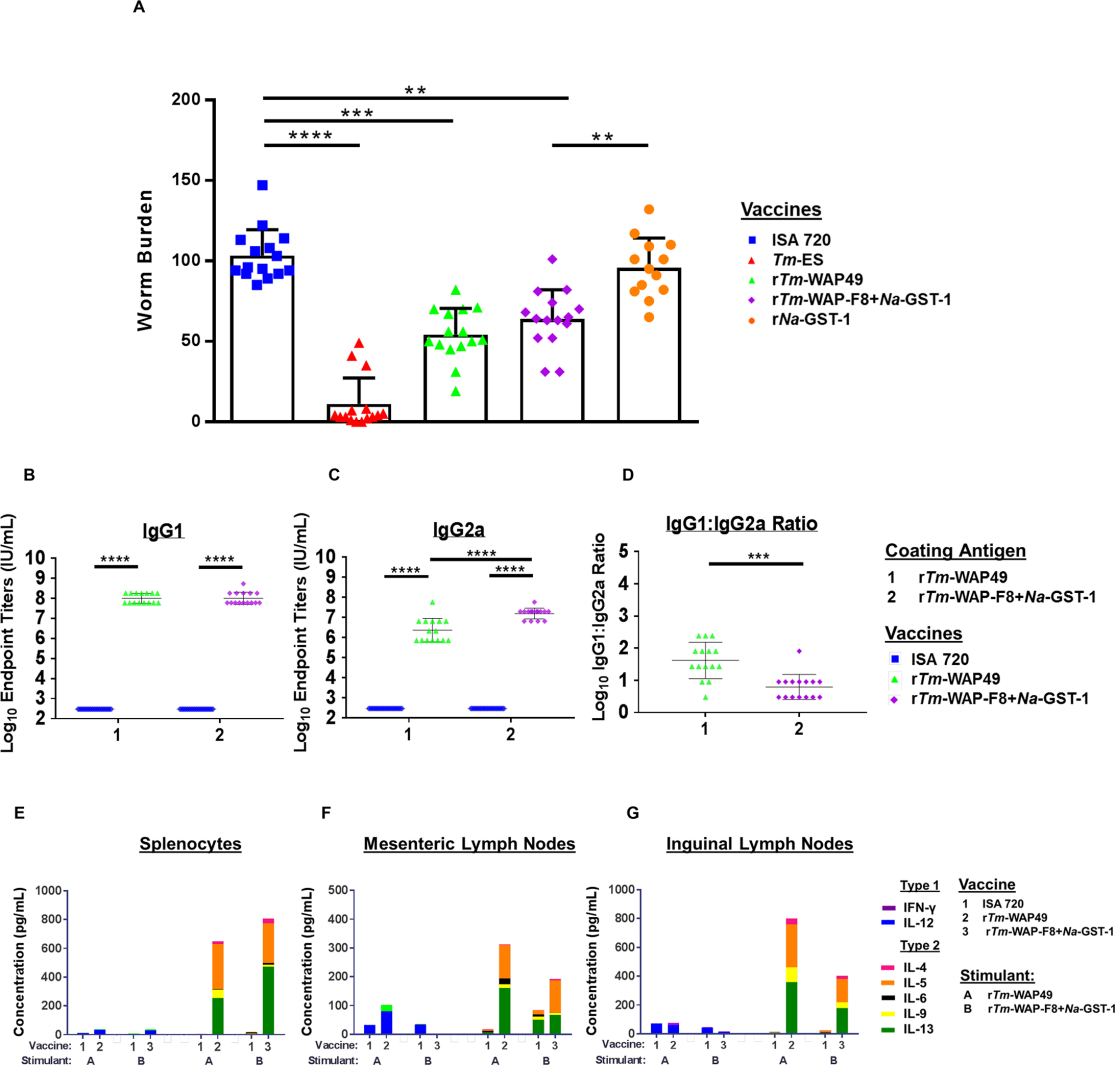
Efficacy and anamnestic responses in mice immunized with r*Tm*-WAP49 or r*Tm*-WAP-F8+*Na*-GST-1. (A) Vaccine efficacy was evaluated on day 15 post-infection by counting of worms in the intestines by microscopy and compared to Montanide ISA 720 control. At the time of sacrifice, anamnestic humoral immune response in terms of end-point serum titers for antigen-specific (B) IgG1 and (C) IgG2a was measured by ELISA and shown as an (D) IgG1 to IgG2a ratio. (E) Splenocytes are shown as individual values for each group (n = 15), and (F) MLNs and (G) ILNs are separately pooled as three groups of five mice (n = 3). Mice in groups injected with Montanide ISA 720 were used as negative controls for each stimulant. Cells receiving no stimulus (media only) were subtracted as background from stimulated cells from corresponding mice. Statistical significance: *p<0.05, **p<0.01 ***p<0.001, ****p<0.0001. Individual cytokines and statistics are shown in S3 Fig.

Next, we compared the efficacy and immune correlates for r*Tm*-WAP-F8+*Na*-GST-1 and r*Tm*-WAP49 immunogens using the same vaccination and challenge schedule as described above. We also included a group of mice vaccinated with r*Na*-GST-1 to account for its potential contribution to protection observed for r*Tm*-WAP-F8+*Na*-GST-1. In comparison to the Montanide ISA 720 adjuvant control, a significant reduction in worm burden was observed in mice vaccinated with r*Tm*-WAP49 (48%, p = 0.000103) or r*Tm*-WAP-F8+*Na*-GST-1 (38%, p = 0.00278), with no significant difference among these two groups. Importantly, vaccination with *Tm*-ES resulted in significant protection, while r*Na*-GST-1 was ineffective yielding no significant reduction in worm burden after challenge. These results suggest that the protective efficacy observed with the r*Tm*-WAP-F8+*Na*-GST-1 against challenge with the *T*. *muris* eggs was related to the WAP fragment 8, and not the *Na*-GST-1 tag (**Fig 5A**).

Immune correlates for the protective efficacy by r*Tm*-WAP49 and r*Tm*-WAP-F8+*Na*-GST-1 included significantly higher antigen-specific IgG1 endpoint titers post-infection compared to that in mice immunized with the Montanide ISA 720 adjuvant alone (**Fig 5B**). However, r*Tm*-WAP-F8+*Na*-GST-1 generated a higher antigen-specific IgG2a response (**Fig 5C**), leading to a significantly lower IgG1:IgG2a ratio relative to that observed in mice vaccinated with r*Tm*-WAP49 (**Fig 5D**).

We also analyzed for post-challenge cellular immune correlates of protection in the spleen, the parasite-draining mesenteric lymph nodes (MLNs), and the vaccine-draining inguinal lymph nodes (ILNs). In mice vaccinated with r*Tm*-WAP-F8+*Na*-GST-1 or r*Tm*-WAP49, restimulation of spleen cells with cognate antigen showed high levels of IL-4, IL-5, and IL-6, indicative of type 2 immune responses (**Fig 5E**). Interestingly, we observed that the IL-13 response was significantly higher for r*Tm*-WAP-F8+*Na*-GST-1 whereas the IL-9 response was significantly higher for r*Tm*-WAP49, relative to each other (**S3A and S3B Fig**). Strong type 2 responses were also observed in the MLNs of mice vaccinated with r*Tm*-WAP-F8+*Na*-GST-1 or r*Tm*-WAP49 **(Fig 5F**), but IL-6 and IL-13 responses were significantly higher in r*Tm*-WAP49 vaccinated mice compared to those vaccinated with r*Tm*-WAP-F8+*Na*-GST-1 (S**3C and S3D Fig**). Finally, in the inguinal lymph nodes (ILNs) of mice vaccinated with r*Tm*-WAP49, we observed significantly higher type 2 immune responses in terms of higher production of cytokines IL-4, IL-5, IL-6, IL-9, and IL-13, compared to that in mice vaccinated with r*Tm*-WAP-F8+*Na*-GST-1, but both these groups of mice exhibited significantly higher type 2 immune responses compared to control group of mice immunized with adjuvant alone (**Fig 5G, S3E and S3F Fig**).

### *Tm*-WAP is the immunodominant component of *Tm*-ES

**Fig 6:**
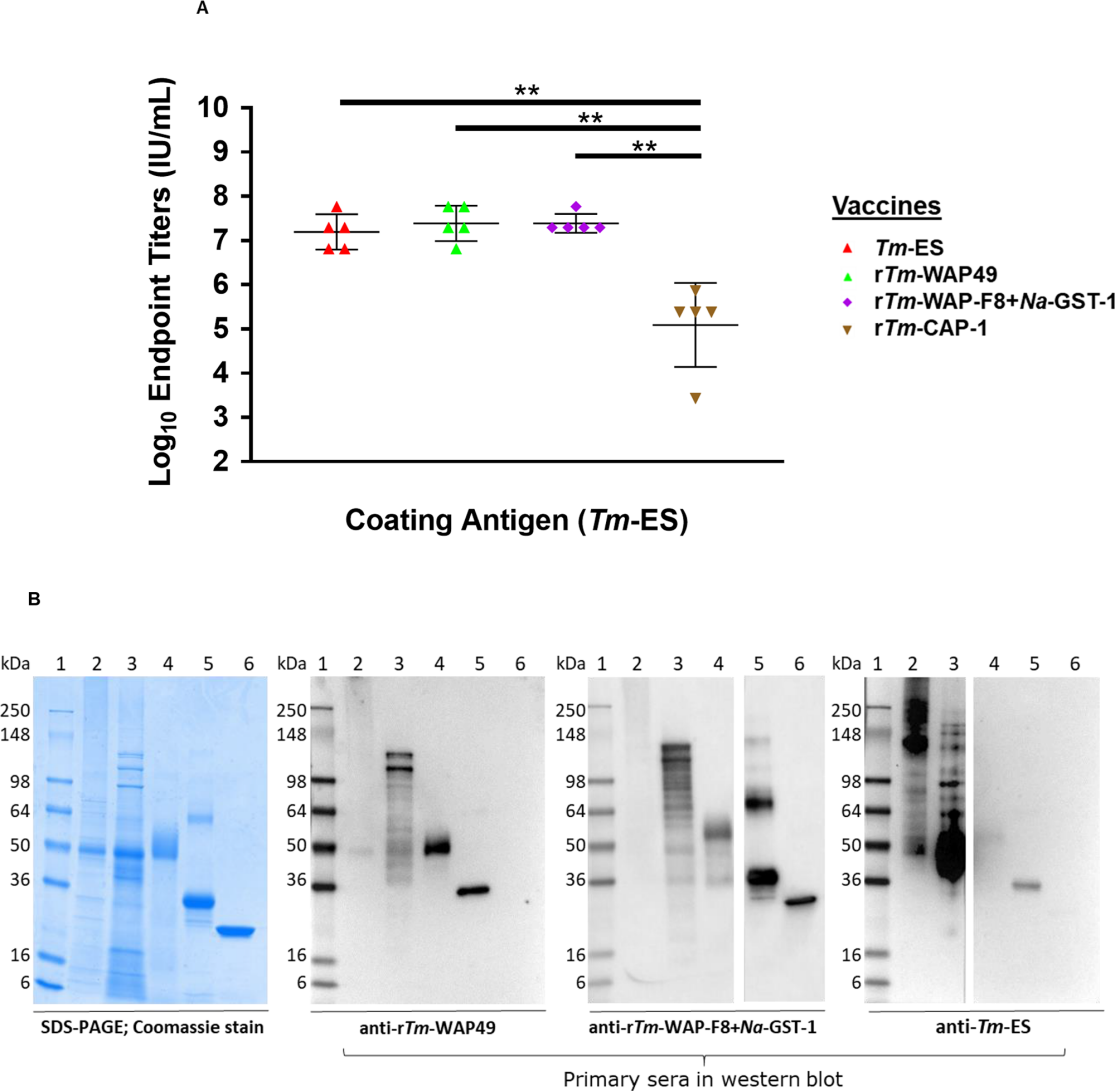
Cross-recognition of native and recombinant *Tm*-WAP and *Tm*-CAP-1 proteins. (A) Serum samples from mice immunized with r*Tm*-WAP49, r*Tm*-WAP-F8+*Na*-GST-1, and r*Tm*-CAP-1, and *Tm*-ES were tested for their IgG antibody recognition of *Tm*-ES by ELISA endpoint titers. Statistical significance: *p<0.05, **p<0.01 ***p<0.001, ****p<0.0001. (B) Western blot analyses for the serological recognition and cross-recognition of r*Tm*-WAP49, r*Tm*-WAP-F8+*Na*-GST-1, and *Tm*-ES. In each panel, the lane numbers on the top represent: (1) SeeBlue pre-stained protein marker; (2) *T*. *muris* adult homogenate; (3) *T*. *muris* ES; (4) Recombinant *Tm*-WAP49 expressed in *P*. *pastoris*; (5) Recombinant *Tm*-WAP-F8+*Na*-GST-1 fusion protein expressed in *E*. *coli*; (6) Recombinant *Na*-GST-1 expressed in *P*. *pastoris*. The SDS-PAGE was performed using 4–20% NuPAGE/MES gel. WB was performed using different mouse anti-sera (1:3,000) as shown under each panel. For WB with anti-*Tm*-ES, the loaded amount of protein was adjusted for lane 2 to 6 as 2.0 μg, 500 ng, 1.0 μg, 400 ng and 400 ng. The nitrocellulose membranes of anti-r*Tm*-WAP-F8+*Na*-GST-1 and anti-*Tm*-ES were cut just prior to ECL substrate reaction between lanes 4–5 and 3–4, respectively, to allow for different length of exposure time to detect the signal.

Serum samples from mice immunized with the various *T*. *muris*-derived immunogens (r*Tm*-WAP49, r*Tm*-WAP-F8+*Na*-GST-1, and r*Tm*-CAP-1) were tested for their antibody recognition of *Tm*-ES (**Fig 6A**). ELISA endpoint titers revealed that both r*Tm*-WAP49 and r*Tm*-WAP-F8+*Na*-GST-1 have significantly greater recognition of *Tm*-ES than *Tm*-CAP-1. Furthermore, antibody recognition of *Tm*-ES by r*Tm*-WAP49 and r*Tm*-WAP-F8+*Na*-GST-1 were the same as *Tm*-ES antisera. We performed western blot analyses of multiple *T*. *muris* products using serum samples from mice vaccinated with r*Tm*-WAP49, r*Tm*-WAP-F8+*Na*-GST-1, or *Tm*-ES (**Fig 6B**). We observed that mouse anti-*Tm*-ES serum originally used for the immunoscreening of the *T*. *muris* cDNA library clearly identified 49kd and 31kd protein bands corresponding to r*Tm*-WAP49 and r*Tm*-WAP-F8+*Na*-GST-1, respectively, and confirming *Tm*-WAP as an immunodominant antigen in the ES products of the *T*. *muris* adult worm. The mouse anti-r*Tm*-WAP49 sera recognized r*Tm*-WAP49 and the WAP fragment within r*Tm*-WAP-F8+*Na*-GST-1, but not the r*Na*-GST-1 tag. Sera from mice immunized with r*Tm*-WAP-F8+*Na*-GST-1 recognized r*Tm*-WAP49 and r*Na*-GST-1. Importantly, sera from mice immunized with either *Tm*-WAP49 or r*Tm*-WAP-F8+*Na*-GST-1 exhibited a similar recognition pattern of *Tm*-ES products, with several bands from 28–148 kDa range, corroborating findings that highly-conserved WAPs may be expressed as several secretory proteins of different molecular weights by both *T*. *muris* [27] *and T*. *trichiura* [24].

### Native *Tm*-WAP protein localizes to the stichosome organ of *T*. *muris*

**Fig 7:**
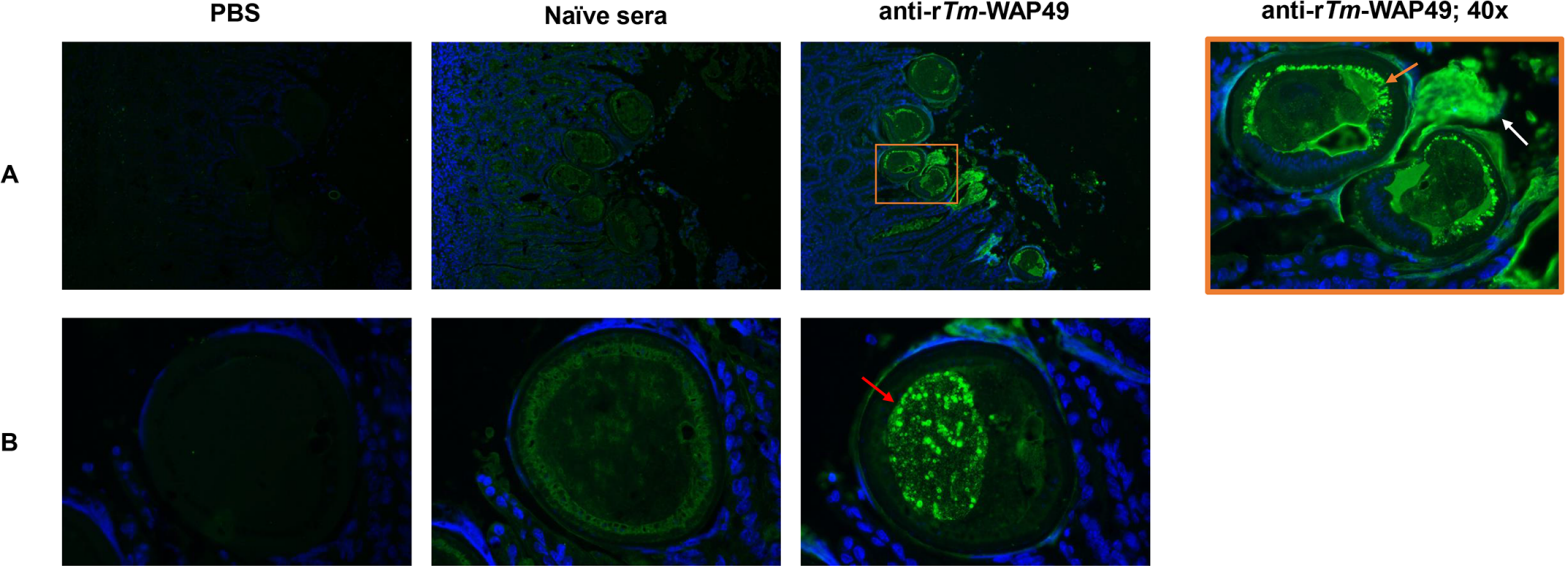
Immunofluorescent staining of *Tm*-WAP proteins expressed in adult *Trichuris muris* stichosome. (A) Cross-sections of murine cecum embedded with multiple adjacent adult *T*. *muris* at 20x magnification. The orange box was imaged at 40x to the right. Embedded *T*. *muris* revealed *Tm*-WAP recognition in a ring pattern adjacent to the cuticle of the helminth (orange arrow) with presumptive *Tm*-WAP secretions into the caecal lumen (white arrow). (B) Sections of embedded *T*. *muris* showing *Tm*-WAP recognition in a distinct granular pattern within a discernable internal structure (red arrow). Primary staining was performed with PBS, naïve sera (1:100), or anti-r*Tm-*WAP49 (1:100). Secondary staining was with FITC-conjugated goat anti-mouse IgG (1:500). DAPI was embedded in the mounting medium. SCID mice were infected with *T*. *muris* for 42 days.

Over the past 20 years, the *Trichuris spp*. stichosome has been of particular interest due to its pathogenic role in facilitating its intercellular existence within the intestinal epithelial layer [6,9,24,35]. To determine whether *Tm*-WAP is stichosome-derived, we used anti-r*Tm*-WAP49 sera to perform fluorescent immunohistochemistry (IHC) of adult *T*. *muris* embedded in the caecal tissue (visualized by DAPI nuclear stain). Tissue cross-sections revealed an anti-*Tm*-WAP staining in the tissue-embedded stichosome of *T*. *muris* (**Fig 7**) with no discernable staining in the posterior, non-stichosome end of the worm. A predominance of the staining occurred in a ring-like, granular pattern (orange arrow) beneath the cuticle of the helminth. Directly adjacent to the outer layer of the helminth cuticle, we observed strong anti-*Tm*-WAP binding (white arrow), suggesting secretion of the WAP protein into the mouse caecal intraluminal space. Of note, in certain cross sections, there were delineated structures with granular anti-*Tm*-WAP staining (red arrow) within the stichosome. A similar pattern of variable distribution of antigen-specific secretory granules was previously described in the stichosome of *T*. *spiralis* [36].

### Recombinant *Tm*-WAP proteins do not elicit antigen-specific IgE response

The generation of antigen-specific IgE antibodies secondary to vaccination or pre-existing in the population would likely impair the protective efficacy of immunization with that specific candidate vaccine [37]. In response to a systemic vaccination these circulating parasite-specific IgE antibodies can lead to a deleterious allergic response, as seen with the hookworm *Na*-ASP-2 vaccine [38]. Serum-specific IgE to total IgE ratio is a clinical indicator of allergen-specific response [39,40]. We observed that vaccination with either r*Tm*-WAP49 or r*Tm*-WAP-F8+*Na*-GST-1 did not lead to any significant serum-specific IgE to total IgE ratio compared to Montanide ISA 720 (**S4A–S4C Fig**). Ratios fell below a cutoff defined by the average values observed analyzing serum samples from control group of mice immunized with PBS. Furthermore, we evaluated mice for serum-specific IgE endpoint titers post-infection to the two recombinant WAPs by ELISA (S4D Fig). While IgE antibodies did react to *Tm*-Lysate, titers against the recombinant WAP proteins remained comparable to the negative control, *Na-*GST-1. Additionally, *Tm*-ES, which contains the immunodominant native *Tm*-WAP, did not induce an IgE response after vaccination nor was *Tm*-ES recognized by IgE antibodies in the sera of infected mice.

## Discussion

With nearly a half-billion people afflicted, trichuriasis is one the most prevalent infectious diseases in the world, and yet there are limited prophylactic and therapeutic options. Using the *T*. *muris* infection of AKR mouse as a surrogate model of *T*. *trichiura* infection in humans, here we describe the identification of a whey acidic protein (WAP) as a highly expressed secretory protein from the stichosome of the worm that is effective in inducing a strong Th2 immunity and significant protection against oral challenge with *T*. *muris* eggs. The recombinant WAP protein (r*Tm*-WAP49) as well as the highly conserved fragment 8 of WAP expressed as a fusion protein with *Na*-GST-1 tag (r*Tm*-WAP-F8+*Na*-GST-1) were equally effective in inducing protective Th2 immunity.

Vaccination with either *Tm*-WAP (r*Tm*-WAP49) or *Tm*-WAP fragment fusion protein (r*Tm*-WAP-F8+*Na*-GST-1) led to a significantly reduced worm burden after *T*. *muris* infection. Interestingly, relative protection after vaccination with r*Tm*-WAP49 was greater but not significantly different from that observed after vaccination with r*Tm*-WAP-F8+*Na*-GST-1.

Antibody recognition of both r*Tm*-WAP49 protein and the fragment r*Tm*-WAP-F8 by *Tm*-ES antisera and the reciprocal recognition of native *Tm*-WAP in *Tm*-ES by anti-r*Tm*-WAP49 and anti-r*Tm*-WAP-F8+*Na*-GST-1 sera, suggest that both candidates, as well as *Tm*-WAP in the *Tm*-ES, contain epitopes with conserved antigenicity. Furthermore, using r*Tm*-WAP49 serum, we localized the native protein to the stichosome organ of *T*. *muris*. Protection generated in mice vaccinated against *Tm*-WAP further supports previous studies suggesting that the stichosome plays a critical role in helminth pathogenicity [41,42]. The homologous stichosome-derived secretory *Trichuris trichiura* WAPs, *Tt*52 and *Tt*95, are thought to be part of a multigene WAP family, due to gene duplication and elongation events. Consistent with this finding in *T*. *trichiura*, multiple proteins from the *T*. *muris* ES are cross-reactive to anti-*Tm*-WAP49 and anti-*Tm*-WAP-F8+*Na*-GST-1 sera by western blot. Furthermore, *Tt*52 and *Tt*95 have been described for their ability to generate pores in a planar lipid bilayer [12,24,25]. Future studies testing the ability of *Tm*-WAP antisera to inhibit pore-formation, preventing stichosome penetration and maintenance in the colonic mucosa would be important to fully realize the vaccine potential of WAP.

Critical cellular mediators of protection against *T*. *muris* identified in our studies are the Th2 cytokines IL-4, IL-9, and IL-13. In this regard, it is well-established that the Th2 cytokine IL-4 is a critical mediator to enhance antibody responses [43], while IL-13 is recognized for its role in promoting worm expulsion through goblet cell expansion, mucin production, and smooth muscle hypercontractility [43,44]. Similarly, IL-9 mediates smooth muscle hypercontractility, as well as tissue mast cell maturation [44,45]. Given the important roles for these Th2 cytokines in the infection process of the worm, our results demonstrating the protective immunity elicited by the recombinant WAP protein, or the highly conserved fragment 8 of the WAP, strongly support their potential as vaccine candidates.

In contrast to the protective efficacy of the WAP, *Tm-*CAP-1, the second highly expressed protein identified in our studies from the excreted-secreted products of *T*. *muris*, was ineffective in inducing strong type 2 immune response as well as protection. The lack of protective efficacy for *Tm*-CAP-1 is in line with our results showing that antisera against this protein do not exhibit ELISA recognition of *Tm*-ES. On the other hand, the antisera from mice immunized with r*Tm*-WAP49 and r*Tm*-WAP-F8+*Na*-GST-1, suggesting that the native *Tm*-WAP protein is the immunodominant and major protective component of *Tm*-ES. This is in contrast to literature reports identifying the CAP-domain family (also known as SCP/TAPS) as a promising vaccine target due to its abundance in the secretome of whipworm and other soil-transmitted helminths [26,27]. Additional studies testing different immunization regimens and/or adjuvant formulations should inform whether or not the CAP-1 protein can induce Th2 immunity and protective efficacy.

Pre-formed IgE antibodies to recombinant *Na*-ASP-2 in vaccine recipients from hookworm endemic regions are thought to have induced generalized urticaria, halting the vaccine’s development [46]. In this regard, after three immunizations with either of the two recombinant WAP proteins we did not observe the parasite-specific IgE to total IgE ratio to be above background levels. Furthermore, *T*. *muris* infection did not induce IgE antibodies against either recombinant WAP protein. Future studies evaluating the potential of the recombinant WAP proteins as vaccine candidates should determine whether natural infection with *T*. *trichiura* generates pre-formed IgE antibodies that recognize the recombinant *Tm*-WAP proteins.

The high degree of amino acid sequence identity for the WAP proteins between *T*. *muris* and *T*. *trichiura* also bodes well for WAP as a strong candidate for vaccine development strategies against *T*. *trichiura*. Further evaluation of the immunogenicity of these vaccine candidates with more potent Th2-promoting adjuvants, such as the alum-based adjuvants, along with optimizing the dose and the route of delivery (e.g. mucosal vs systemic), may augment protection. Additional *Tm*-ES derived antigens, such as those recently reported by Shears et al. [34], may be required in conjunction to *Tm*-WAP to achieve the same near-sterile immunity generated by *Tm*-ES vaccination.

In summary, here we report the identification of a novel immunogenic whipworm protein, *Tm*-WAP, a secretory, stichosome-derived protein. We have developed two recombinant derivatives of *Tm*-WAP, r*Tm*-WAP-F8+*Na*-GST-1 and r*Tm*-WAP49, which are both protective in the AKR animal model. Furthermore, we demonstrate that both candidates generate a strong type 2 immune response, similar to the highly protective *Tm*-ES. Future evaluations will require verification that *Na*-GST-1 fused with *Tm*-WAP-F8 retains protective efficacy as an individual candidate against hookworm, supporting the potential to developing pan-anthelminthic vaccine [29,47,48].

## Methods

### Ethics statement

All animal procedures were conducted in accordance with Baylor College of Medicine Institutional Animal Care and Use Committee (IACUC) approved protocol AN-6297 in compliance with the Animal Welfare Act, PHS Policy, and other Federal statutes and regulations relating to animals and experiments involving animals.

### Generation of T. muris excretory and secretory (ES) products and their anti-sera

The *T*. *muris* ES products were obtained from the overnight culture of *T*. *muris* adult worms isolated from laboratory maintained STAT6/KO mice based on the established protocol [10,13,17]. The concentrated *T*. *muris* ES products containing the stichosome-secreted proteins (100 μg/mouse) formulated with Montanide ISA 720 were used to immunize AKR mice subcutaneously three times at 2 weeks interval. The antisera were obtained from immunized mice 10 days after the last immunization.

### Immunoscreening of the *T*. *muris* adult cDNA library

Mouse anti-ES sera were used to immunoscreen *T*. *muris* adult cDNA library to identify immunodominant antigens based on the methods described previously [49]. Briefly, 5x10^4^ plaques of the cDNA library made from *T*. *muris* adult worm were plated on each LB agar plate. The expression of *T*. *muris* adult proteins was induced and transferred by covering each plate with a 10 mM IPTG soaked nitrocellulose membrane. The blotted membranes were incubated with 1:2,000 dilutions of mouse anti-ES sera and then reacted with HRP-conjugated goat anti-mouse IgG (1:5000) (Invitrogen, USA). The putative positive clones were scored and picked for secondary screening with the same reagents until a single positive clone was obtained.

The DNA sequences of positive clones were obtained by double strand DNA sequencing using vector flanking primers, T3 and T7 promoter. Nucleotide and deduced amino acid sequences were compared to existing sequences by BLAST searching in the GenBank (http://www.ncbi.nlm.nih.gov) and in the *T*. *muris* reference genome on WormBase ParaSite (http://parasite.wormbase.org/Trichuris_muris_prjeb126/). MUSCLE 3.8.31 was used for multiple sequence alignment while PhyML 3.1 for phylogeny and TreeDyn 198.3 was used for phylogenetic tree rendering using one click analysis mode online at http://www.phylogeny.fr [50,51].

### Expression and purification of *Tm*-WAPs and *Tm*-CAP-1

DNA encoding for the *Tm*-WAP49, comprising 70% of the immunodominant *Tm*-WAP (amino acids 24–393), was amplified without signal peptide from the total *T*. *muris* adult cDNA using forward (5’-GCGAATTCGCTAAAATAGGTTCATGTCC-3’) and reverse (5’-ATGCGGCCGCTCAATGATGATGATGATGATGCTCGGCAGTGCTGTCGTCTCGGT-3’) primers and subcloned into yeast expression vector pPICZαA (Invitrogen, USA) using EcoRI and XbaI restriction enzyme sites. The correct open reading frame (ORF) was confirmed by sequencing using the vector flanking primers corresponding to the regions encoding the α-factor and 3’AOX1. The recombinant plasmids were linearized following digestion with SacI and transformed into *P*. *pastoris* X33 strain by electroporation according to the manufacturer’s instructions (Invitrogen, USA). Transformants were selected on zeocin-resistant YPD plates. The expression of recombinant *Tm*-WAP49 with 6His-tag at C-terminus were induced in medium with methanol in 10 L fermentation for 96 hours. The expressed recombinant *Tm*-WAP49 was purified with immobilized metal affinity chromatography (IMAC), as previously described [30].

For cloning *Tm*-WAP-F8+*Na*-GST-1, DNA coding for a *Tm*-WAP repeat fragment 8, the most conserved (69–96%) of the 10 repeats of *Tm*-WAP, was synthesized by GenScript. *Tm*-WAP-F8 was then fused with DNA coding for *Na*-GST-1 at N-terminus, a hookworm vaccine antigen with GST function that helps the solubilization of fusion protein [29,52] and cloned into pET41a by using NdeI/NdeI/XhoI sites. The *Tm*-WAP-F8+*Na*-GST-1 was expressed as soluble recombinant fusion protein in *E*. *coli* BL21 under induction of 1 mM IPTG and purified with immobilized metal affinity chromatography.

For cloning *Tm*-CAP-1, the DNA encoding for *Tm*-CAP-1 (cysteine-rich secretory proteins, antigen 5, and pathogenesis-related 1 protein of *Trichuris muris*), without N-terminus signal peptide and C-terminus GPI anchor, was amplified from *T*. *muris* adult cDNA using forward (*Tm*-CAP-1-F2: GACATATGGCACCACTTACGGTCCCCAT) and reverse (*Tm*-CAP-1-R2: TCGCGGCCGCTCAATGATGATGATGATGATGTTCTCTTAGGAGAAGAACGT) primers and subcloned into *E*. *coli* expression vector pET41A (Novagen, USA) using NedI/NotI sites. The sequencing-confirmed correct recombinant plasmid was transformed into *E*. *coli* BL21 (DE3) and the recombinant *Tm*-CAP-1 protein (r*Tm*-CAP-1) was expressed under 1mM IPTG induction at 30°C overnight. The r*Tm*-CAP-1 was expressed as insoluble inclusion body and solubilized in 8M urea. The r*Tm*-CAP-1 with 8 his-tag at C-terminus was purified with IMAC. The purified urea-denatured r*Tm*-CAP-1 was refolded in the refolding buffer (250 mM Arginine, 150mM NaCl, 50mM Tris, pH 8.0).

### Electrophoresis and immunoblotting of recombinant *Tm*-WAP proteins

Samples were separated by 4–20% NuPAGE/MES gel (Thermo Fisher Scientific, USA) and either immediately stained with Coomaisse Blue for imaging analysis or transferred onto PVDF membrane (Thermo Fisher Scientific, USA). PVDF membranes were blocked with 5% (w/v) skim milk powder in PBST (PBS +0.05% Tween-20), and then incubated with sera (1:3,000) from immunized mice (r*Tm*-WAP49, r*Tm*-WAP-F8+*Na*-GST-1 or *Tm*-ES. HRP-conjugated goat anti-mouse IgG (Invitrogen, USA) was used as a secondary antibody, followed by ECL substrate (GE Healthcare, USA) to develop antibody bound bands. The nitrocellulose membranes of anti-r*Tm*-WAP-F8+*Na*-GST-1 and anti-*Tm*-ES were cut just prior to ECL substrate reaction between lanes 4–5 and 3–4, respectively, to allow for different length of reaction. Both *T*. *muris* adult homogenate and *Tm*-ES were used at 10 μg for SDS-PAGE and 2.0 μg for western blot. r*Tm*-WAP49 was used at 2 μg for SDS-PAGE and 200 ng for western blot. Both r*Tm*-WAP-F8+*Na*-GST-1 and r*Na*-GST-1 were used at 2 μg for SDS-PAGE and 100 ng for western blot.

### Immunofluorescence staining of mouse cecum

Immunofluorescent staining for *Tm*-WAP in adult *Trichuris muris* was done following the M.O.M. Immunodetection Kit (Vector Labs, USA). In brief, SCID mice were sacrificed on day 42 post *T*. *muris* infection. Intestinal tissue with worms embedded were fixed in paraffin and sectioned. Primary staining was performed with PBS, naïve sera (1:100), or anti-r*Tm-*WAP49 (1:100). Secondary staining was with FITC-conjugated goat anti-mouse IgG (1:500). DAPI was embedded in the mounting medium.

### Immunization

The protein concentration of each antigen (r*Tm*-WAP49; r*Tm*-WAP-F8+*Na*-GST-1; r*Tm*-CAP-1; *Tm*-ES) was measured using BCA (Thermo Fisher Scientific, USA) and determined to be endotoxin free (<0.88 EU/ mg) by Charles River Endosafe-PTS system (Charles River, USA). Vaccine antigens were buffered in sterile, ultrapure grade phosphate-buffered saline (PBS) (VWR, USA) and emulsified with Montanide ISA 720 at 30:70 aqueous to oil based on volume, as previously described [31]. *Tm*-ES was administered at 100 μg of antigen per dose, while r*Tm*-WAP49, r*Tm*-WAP-F8+*Na*-GST-1, and r*Tm*-CAP-1 were administered at 100 μg of antigen per dose. Vaccinations were given at 100 μL per dose, subcutaneously at the lateral tail base.

### *T*. *muris* parasite and mouse model

*T*. *muris* E (Edinburgh) isolate, kindly provided by Dr. Joe Urban from the USDA, were maintained in susceptible STAT6K/O mice. At day 15 post-infection (p.i.) (42 days after initial vaccination), adult worms were gently extracted by forceps from the caecum of sacrificed mice. Day 15 p.i., which corresponds to the L2/L3 larvae [18], was selected due our observed peak intensity of infection in AKR mice. *T*. *muris* eggs collected from cultured *T*. *muris* adult worms were washed in sterile water and centrifuged at 500xg for 5 minutes and supernatant debris was removed from pelleted eggs. Collected eggs were stored in distilled water in culture flasks (VWR, USA) and allowed to embryonate at room temperature in the dark for 8 weeks. 300 embryonated eggs were separated into 100 μL aliquots. Each aliquot was counted by light microscopy to verify 300 ± 10 embryonated eggs.

6-7-week-old male AKR mice were purchased from Jackson Labs (USA) with 5 mice per group for immunogenicity and 13–15 mice per group for efficacy studies. Mice undergoing challenge were infected by oral gavage of 300 embryonated eggs in 100 ul of diH2O. Worm burden and anamnestic immunological response were assessed at day 15 p.i. Blood collection at various time points was collected by tail bleeding and the terminal bleeds were collected by cardiac puncture at the time of sacrifice. Mouse spleen, mesenteric lymph nodes, and inguinal lymph nodes were collected at the time of sacrifice, and immediately resuspended in a single-cell suspension for immunological analysis.

### Antibody analysis

Sera was isolated from blood by centrifugation in serum-separating tube (Sarstedt, Germany) at 10,000 g for 5 minutes and stored at -80°C until assay. Samples were evaluated for antigen-specific horseradish peroxidase (HRP)-conjugated immunoglobulins IgG (Lifespan Biosciences, USA), IgG1 (Lifespan Biosciences, USA), IgG2a (Lifespan Biosciences, USA), and IgE (SouthernBiotech, USA) by a modified indirect enzyme-linked immunosorbent assay (ELISA), as described previously [53]. Antigen coating concentrations of r*Tm*-WAP49 (0.375 μg/mL), r*Tm*-WAP-F8+*Na*-GST-1 (0.375 μg/mL), r*Tm*-CAP-1 (0.375 μg/mL), r*Na*-GST-1 (0.375 μg/mL), *Tm*-ES (0.75 μg/mL), or *Tm*-Lysate (0.75 μg/mL) were selected by pretested optimal signal/noise ratio. The absorbance was measured by dual wavelength analysis at test wavelength of 450 nm minus the reference wavelength of 630 nm using a spectrophotometer (BioTek, USA).

### Cytokine analysis

Spleens, mesenteric lymph nodes (MLNs), and inguinal lymph nodes (ILNs) were obtained from mice two weeks after the third immunization (immunogenicity groups) or at additional 15 days after a subsequent challenge infection (efficacy groups). Tissues were disassociated into a single-cell suspension using a 100 μm cell strainer and resuspended in complete RMPI medium (RPMI 1640 with L-glutamine [VWR, USA], 10% heat inactivated FBS [VWR, Radnor, PA], and 1x pen/strep solution [Thermo Fisher Scientific, USA]). After a 5-minute centrifugation (300 x g), splenocytes were resuspended in 2 mL of ACK lysis buffer (Thermo Fisher Scientific, USA) for 5 minutes to remove red blood cells. After centrifugation cells were resuspended in complete RPMI media and incubated for 2 hours in a 37°C + 5% CO_2_ incubator before counting the number of live cells using a Cellometer Auto 2000 Cell Viability Counter (Nexelcom, USA). To have sufficient cells for multiple cytokine assays, MLNs and ILNs, were separately pooled into 3 sets of 5 mice per tissue.

Cells were seeded in a 96-well U-bottom culture plate (Corning, USA) at 1x10^6^ cells per well in 250 μl medium and restimulated with either r*Tm*-WAP49 (10 μg/mL), r*Tm*-WAP-F8+*Na*-GST-1 (10 μg/mL), r*Tm*-CAP-1 (10 μg/mL), r*Na*-GST-1 (10 μg/mL), or *Tm*-ES (50 μg/mL) at 37°C, 5% CO2 for 72 hours. An unstimulated, media only negative control and a positive control of 20 ng/mL PMA and 1 μg/mL Ionomycin were performed concurrently. After 72 hours the cells were pelleted by centrifugation at 300 x g for 5 min and the supernatants were collected for measuring cytokine production. Concentrations of different stimulants and duration of stimulation were selected based on optimal pretested antigen to negative control ratio. Initially supernatant samples were tested using a custom Bio-Plex Pro Mouse Cytokine 10-plex kit (Bio-Rad, USA) for levels of IL-4, IL-5, Il-6, IL-9, IL-10, IL-12(p70), IL-13, IL-17A, IFN-γ and TNF-α. To evaluate accessory cytokines/chemokines associated with protection, subsequent experiments used the expanded Bio-Plex Pro Mouse Cytokine 23-plex kit, which including the original 10 cytokines, added Eotaxin, G-CSF, GM-CSF, IL-1α, IL-1β, IL-2, IL-3, IL-12 (p40), KC, MCP-1 (MCAF), MIP-1α, MIP-1β, and Rantes. To increase the sensitivity of the experiment, the kit was used in combination with DA-Bead plates (Curiox Biosystems, Singapore), as previously described [54]. Samples were run on a Luminex Magpix multiplex reader according to manufacturer's recommendations (Luminex, USA). Raw Luminex data were analyzed using the Bio-Plex Manager 6.0 software and plotted in GraphPad Prism 7.04 or R programming language. Sensitivity cutoff values for the detection of different cytokines were dependent on the included standards for each lot number of the Bio-Rad Bio-Plex kit. Cytokine values from media-only stimulated cells were deemed background and thus were subtracted from the values of antigen restimulated samples.

### Graphical presentation and statistical analysis

Figs 2, 3, 4 and 5 and S4 Fig were generated in GraphPad 7.04. Non-parametric analysis was deemed necessary to compare groups, thus Kruskal-Wallis H test with Dunn’s Multiple Comparisons and Mann-Whitney U test were preformed using GraphPad Prism version 7.04 for Windows, GraphPad Software, La Jolla California USA (www.graphpad.com).

For S1, S2 and S3 Figs, data was loaded into R programming language environment version 3.4.3 using Rstudio version 1.1.423 (http://www.rstudio.com/). Readxl package was used to import the data from the spreadsheet (https://CRAN.R-project.org/package=readxl). Tidyverse package (version 1.2.1) was used to transform the imported data for the analysis (https://CRAN.R-project.org/package=tidyverse). Mean for each cytokine in a group was calculated by bootstrapping using smean.cl.boot function with default setting of Hmisc package (version 4.1–1) (https://CRAN.R-project.org/package=Hmisc) Wilcox test for each cytokine between groups was performed using coin package (version 1.2–2) and effect size was calculated using Cliff delta using effsize package [55,56]. Plot of the calculated results was generated using ggplot2 package (version ggplot2 2.2.1) [57].

For all graphs, p-values were represented as *, **, ***, and ****, corresponding to p-values of <0.05, <0.01, <0.001, and <0.0001, respectively.

## Supporting information

S1 Fig Pre-challenge cytokine profile in the supernatants of splenocytes from AKR mice immunized with *Tm*-ES, r*Tm*-WAP-F8+*Na*-GST-1, or r*Tm*-CAP-1. Splenocytes from mice in each group were stimulated with media or cognate antigen, (A) *Tm*-ES, (B) r*Tm*-WAP-F8+*Na*-GST-1, or (C) r*Tm*-CAP-1 for 72 hours and the cytokines were detected using a 10-plex assay kit. Mice in groups injected with Montanide ISA 720 were used as negative controls for each coating antigen. Cells receiving no stimulus (media only) were subtracted as background from stimulated cells from corresponding mice. Values from duplicate wells for each treatment/stimulation were averaged for individual cytokines for the 5 mice in each group to calculate statistical significance. Data are presented as means ± 95% confidence interval. Statistical significance: *p<0.05, **p<0.01 ***p<0.001, ****p<0.0001. Effect size: d<0.2, d<0.5, d<0.8, and d>0.8, correspond to N (negligible), S (small), M (medium), and L (large), respectively.
(TIF)

S2 Fig Post-challenge cytokine profile in the supernatants of splenocytes from AKR mice immunized with *Tm*-ES, r*Tm*-WAP-F8+*Na*-GST-1, or r*Tm*-CAP-1 and challenged with *T*. *muris* 15 days after the last dosing. Splenocytes from mice in each group were stimulated with media or cognate antigen, (A) *Tm*-ES, (B) r*Tm*-WAP-F8+*Na*-GST-1, or (C) r*Tm*-CAP-1 for 72 hours and the cytokines were detected using a 10-plex assay kit. Mice in groups injected with Montanide ISA 720 were used as negative controls for each coating antigen. Cells receiving no stimulus (media only) were subtracted as background from stimulated cells from corresponding mice. Values from duplicate wells for each treatment/stimulation were averaged for individual cytokines for the 13–15 mice in each group to calculate statistical significance. Data are presented as means ± 95% confidence interval. Statistical significance: *p<0.05, **p<0.01 ***p<0.001, ****p<0.0001. Effect size: d<0.2, d<0.5, d<0.8, and d>0.8, correspond to N (negligible), S (small), M (medium), and L (large), respectively.
(TIF)

S3 Fig Post-challenge analyses of cytokine profile in the supernatants of cells from AKR mice immunized with r*Tm*-WAP-F8+*Na*-GST-1 or r*Tm*-WAP49 and challenged with *T*. *muris*. Cytokines detected in the supernatants of (A, B) splenocytes, (C, D) mesenteric lymph nodes (MLNs), or (E, F) inguinal lymph nodes (ILNs) after stimulation with media only or cognate antigen, r*Tm*-WAP-F8+*Na*-GST-1 (10 μg/mL) or r*Tm*-WAP49 (10 μg/mL) for 72 hours were detected using a 23-plex assay kit. Mice in groups injected with Montanide ISA 720 were used as negative controls for each coating antigen. Cells receiving no stimulus (media only) were subtracted as background from stimulated cells from corresponding mice. Values from duplicate wells for each treatment/stimulation were averaged for individual cytokines for the 15 mice in each group to calculate statistical significance. Data are presented as means ± 95% confidence interval. Statistical significance: *p<0.05, **p<0.01 ***p<0.001, ****p<0.0001. Effect size: d<0.2, d<0.5, d<0.8, and d>0.8, correspond to N (negligible), S (small), M (medium), and L (large), respectively.
(TIF)

S4 Fig Humoral allergenicity of rTm-WAP49 and rTm-WAP-F8+Na-GST-1 in a murine model. (A) Serum-specific IgE was measured by ELISA and (B) total IgE concentration was determined on a standard curve and shown as a (C) serum-specific IgE to total IgE ratio (n = 15 per group). Mice vaccinated with Montanide ISA 720 were used as negative controls for each coating antigen. Cutoff (black dashed line) was defined by the average ratio induced by a PBS vaccinated group. (D) IgE antibody generation against recombinant *Tm*-WAP proteins after infection were measured by endpoint serum titers of mice by ELISA. Recombinant *Na*-GST-1 was used as a negative control and *Tm*-Lysate as a positive control. Statistical significance: *p<0.05, **p<0.01 ***p<0.001, ****p<0.0001.
(PDF)
