# Supplementary figures and images for: Trichuris muris whey acidic protein induces type 2 protective immunity against whipworm
Source: PLoS Pathog. 2018 Aug 28;14(8):e1007273. doi: 10.1371/journal.ppat.1007273 (PMC6130879; doi:10.1371/journal.ppat.1007273)

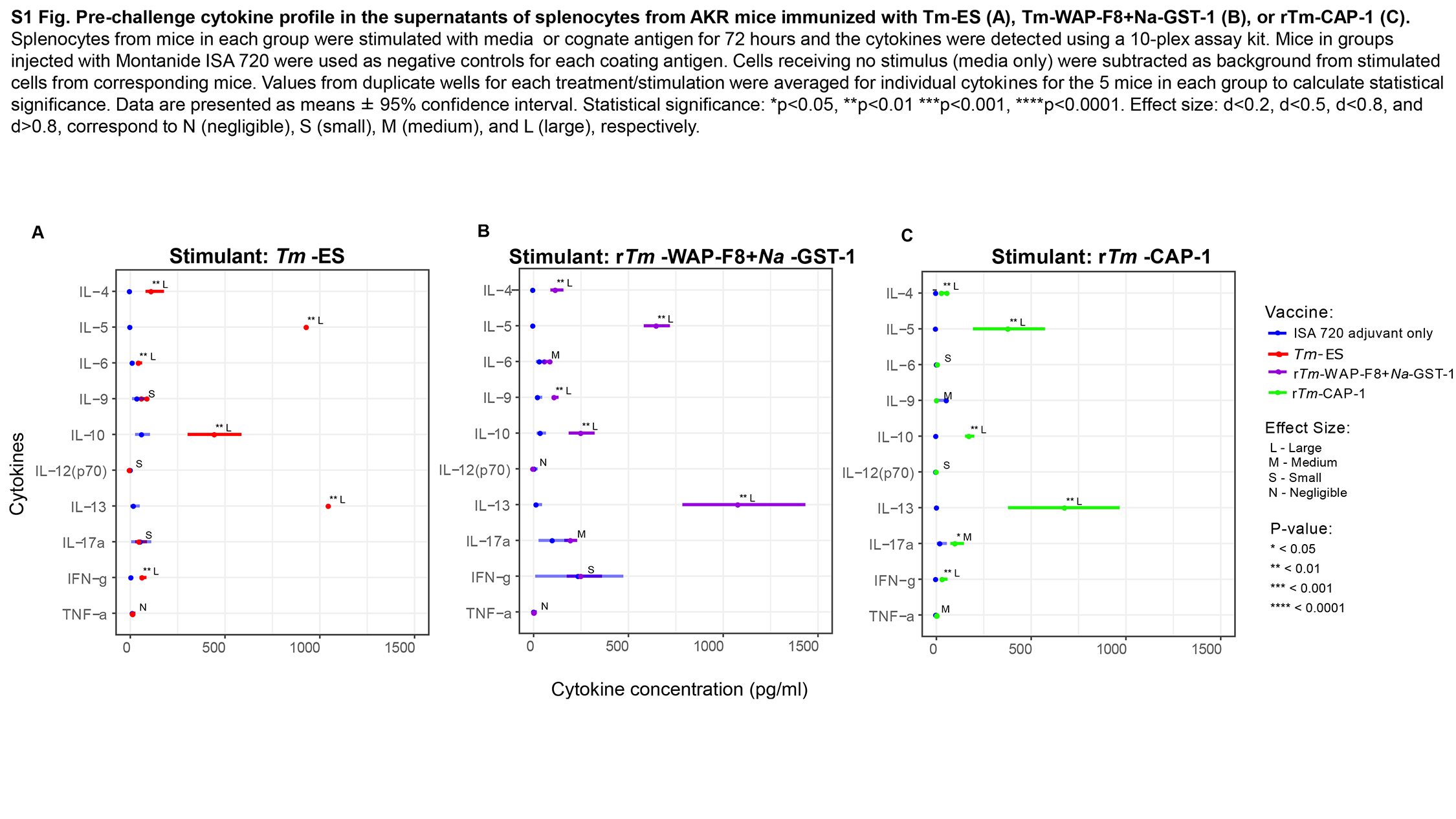

Supplement: S1 Fig — Splenocytes from mice in each group were stimulated with media or cognate antigen, (A) Tm-ES, (B) rTm-WAP-F8+Na-GST-1, or (C) rTm-CAP-1 for 72 hours and the cytokines were detected using a 10-plex assay kit. Mice in groups injected with Montanide ISA 720 were used as negative controls for each coating antigen. Cells receiving no stimulus (media only) were subtracted as background from stimulated cells from corresponding mice. Values from duplicate wells for each treatment/stimulation were averaged for individual cytokines for the 5 mice in each group to calculate statistical significance. Data are presented as means ± 95% confidence interval. Statistical significance: *p<0.05, **p<0.01 ***p<0.001, ****p<0.0001. Effect size: d<0.2, d<0.5, d<0.8, and d>0.8, correspond to N (negligible), S (small), M (medium), and L (large), respectively. (TIF) [file ppat.1007273.s001.tif]

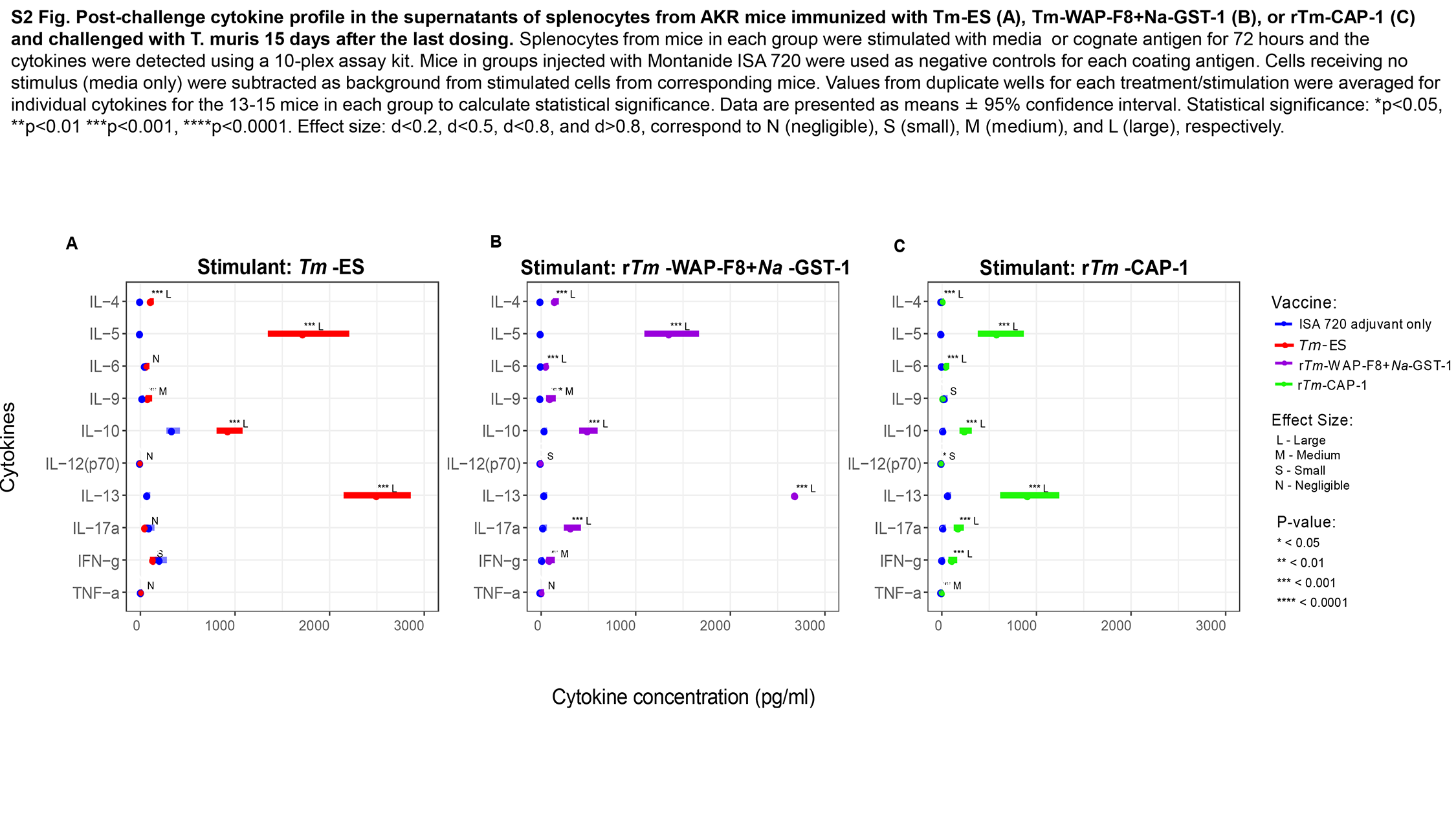

Supplement: S2 Fig — Splenocytes from mice in each group were stimulated with media or cognate antigen, (A) Tm-ES, (B) rTm-WAP-F8+Na-GST-1, or (C) rTm-CAP-1 for 72 hours and the cytokines were detected using a 10-plex assay kit. Mice in groups injected with Montanide ISA 720 were used as negative controls for each coating antigen. Cells receiving no stimulus (media only) were subtracted as background from stimulated cells from corresponding mice. Values from duplicate wells for each treatment/stimulation were averaged for individual cytokines for the 13–15 mice in each group to calculate statistical significance. Data are presented as means ± 95% confidence interval. Statistical significance: *p<0.05, **p<0.01 ***p<0.001, ****p<0.0001. Effect size: d<0.2, d<0.5, d<0.8, and d>0.8, correspond to N (negligible), S (small), M (medium), and L (large), respectively. (TIF) [file ppat.1007273.s002.tif]

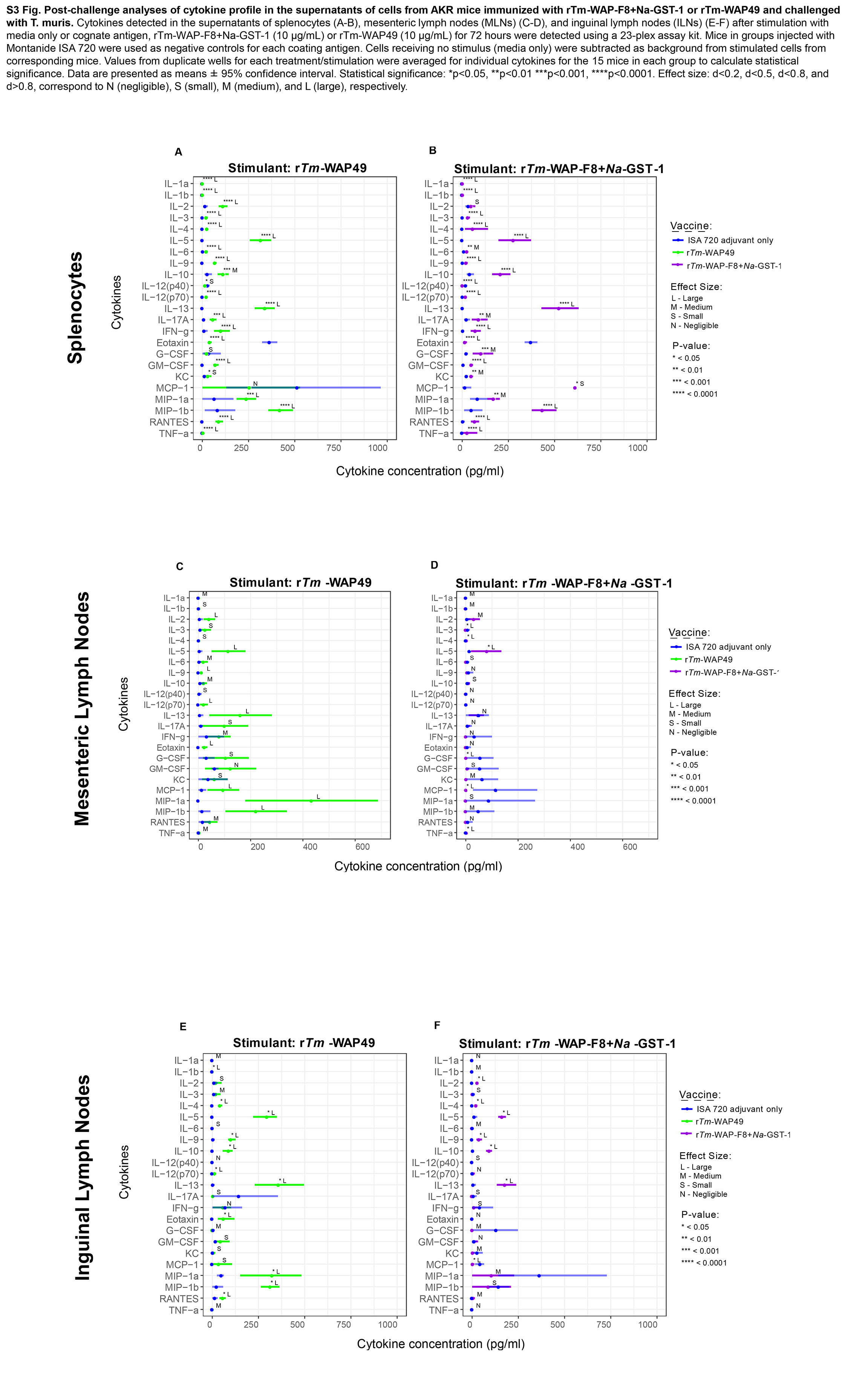

Supplement: S3 Fig — Cytokines detected in the supernatants of (A, B) splenocytes, (C, D) mesenteric lymph nodes (MLNs), or (E, F) inguinal lymph nodes (ILNs) after stimulation with media only or cognate antigen, rTm-WAP-F8+Na-GST-1 (10 μg/mL) or rTm-WAP49 (10 μg/mL) for 72 hours were detected using a 23-plex assay kit. Mice in groups injected with Montanide ISA 720 were used as negative controls for each coating antigen. Cells receiving no stimulus (media only) were subtracted as background from stimulated cells from corresponding mice. Values from duplicate wells for each treatment/stimulation were averaged for individual cytokines for the 15 mice in each group to calculate statistical significance. Data are presented as means ± 95% confidence interval. Statistical significance: *p<0.05, **p<0.01 ***p<0.001, ****p<0.0001. Effect size: d<0.2, d<0.5, d<0.8, and d>0.8, correspond to N (negligible), S (small), M (medium), and L (large), respectively. (TIF) [file ppat.1007273.s003.tif]
