# Supplementary material for: Trichuris muris whey acidic protein induces type 2 protective immunity against whipworm
Source: PLoS Pathog. 2018 Aug 28;14(8):e1007273. doi: 10.1371/journal.ppat.1007273 (PMC6130879; doi:10.1371/journal.ppat.1007273)

S4 Fig. Humoral allergenicity of rTm-WAP49 and rTm-WAP-F8+Na-GST-1 in a murine model.

(A) Serum-specific IgE was measured by ELISA and (B) total IgE concentration was determined on a standard curve and shown as a (C) serum-specific IgE to total IgE ratio (n = 15 per group). Mice vaccinated with Montanide ISA 720 were used as negative controls for each coating antigen. Cutoff (black dashed line) was defined by the average ratio induced by a PBS vaccinated group. (D) IgE antibody generation against recombinant *Tm*-WAP proteins after infection were measured by endpoint serum titers of mice by ELISA. Recombinant *Na*-GST-1 was used as a negative control cut-off (black dotted line) and *Tm*-Lysate as a positive control. Statistical significance: *p<0.05, **p<0.01 ***p<0.001, ****p<0.0001.

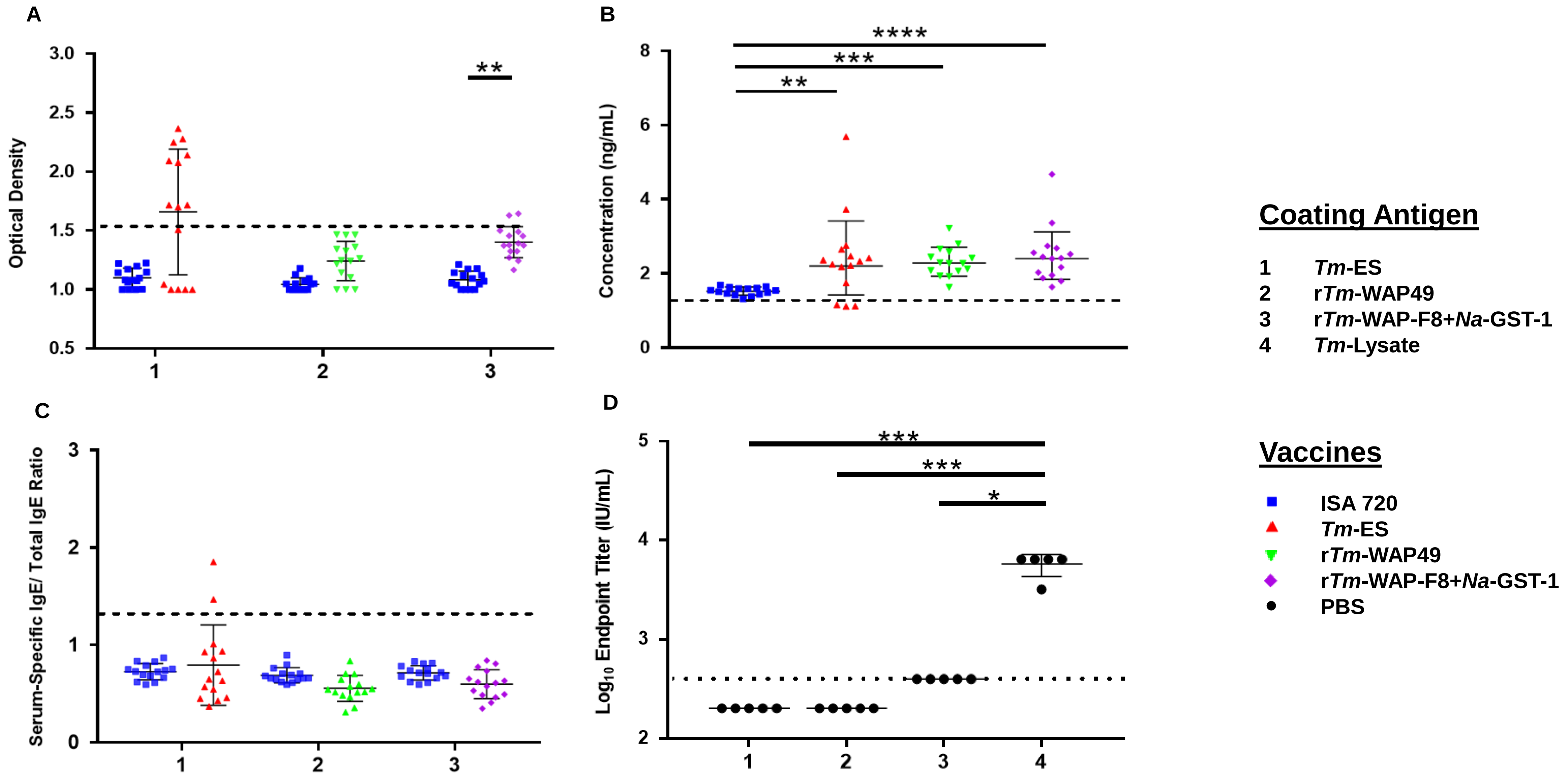

Supplement: S4 Fig — (A) Serum-specific IgE was measured by ELISA and (B) total IgE concentration was determined on a standard curve and shown as a (C) serum-specific IgE to total IgE ratio (n = 15 per group). Mice vaccinated with Montanide ISA 720 were used as negative controls for each coating antigen. Cutoff (black dashed line) was defined by the average ratio induced by a PBS vaccinated group. (D) IgE antibody generation against recombinant Tm-WAP proteins after infection were measured by endpoint serum titers of mice by ELISA. Recombinant Na-GST-1 was used as a negative control and Tm-Lysate as a positive control. Statistical significance: *p<0.05, **p<0.01 ***p<0.001, ****p<0.0001. (PDF) [file ppat.1007273.s004.pdf]
